# Impact of SARS-CoV-2 envelope protein mutations on the pathogenicity of Omicron XBB

**DOI:** 10.1038/s41421-023-00575-7

**Published:** 2023-07-28

**Authors:** Yi Wang, Xiaoyan Pan, Hongying Ji, Xiaoli Zuo, Geng-Fu Xiao, Jia Li, Lei-Ke Zhang, Bingqing Xia, Zhaobing Gao

**Affiliations:** 1 Stake Key Laboratory of Drug Research, Shanghai Institute of Materia Medica, Chinese Academy of Sciences, Shanghai, China; 2 University of Chinese Academy of Sciences, Beijing, China; 3 State Key Laboratory of Medical Neurobiology, Institutes of Brain Science, Fudan University, Shanghai, China; 4 State Key Laboratory of Virology, Wuhan Institute of Virology, Center for Biosafety Mega-Science, Chinese Academy of Sciences, Wuhan, Hubei China; 5 Department of Pharmacology, The Key Laboratory of Neural and Vascular Biology, Ministry of Education, The Key Laboratory of New Drug Pharmacology and Toxicology, Hebei Medical University, Shijiazhuang, Hebei China; 6 Zhongshan Institute for Drug Discovery, Shanghai Institute of Materia Medica, Chinese Academy of Science, Shanghai, China

**Keywords:** Necroptosis, Mechanisms of disease

Dear Editor,

There was a surge of new emergent Omicron variants when the restrictions that were used to quash the virus’s spread were dismantled. Advantageous subvariants had distinct transmission, neutralization and immune escape capabilities. Mutations in the viral spike (S) protein were demonstrated to be responsible for immune escape and enhanced transmission^1^. In comparison with the original strain and other variants, the pathogenicity of Omicron variants was milder^2^. However, it is worth noting that BA.5 infection has shown an increased rate of recovery positivity and an increased proportion of infections that were “symptomatic”^3^. These phenomena remind us to be alert to the change in pathogenicity.

The envelope protein of SARS-CoV-2 (2-E) forms a homopentameric channel that is important for viral virulence^4^. Our previous studies indicated that the 2-E channel is sufficient to induce cell death and even cause acute respiratory distress syndrome (ARDS)-like damage in vivo^4^. Furthermore, T9I, a single high-frequency mutation of 2-E protein in Omicrons, was identified to reduce virus replication and virulence by altering channel function^5^. To further understand the potential contribution of 2-E mutations to pathogenicity, we measured the cell lethality of 2-E spontaneous mutations with a frequency ≥0.01% in five VOCs (Alpha, Beta, Gamma, Delta, and Omicron) up to October 2022 and analyzed the correlation between cell lethality, frequency and clinical severity.

Based on the National Genomics Data Center (NGDC), there are 92 2-E mutations with a frequency ≥0.01% in the five VOCs (Supplementary Fig. S1a and Table S1). Omicron retained 31 mutations that emerged from the early 4 VOCs and gained 7 new mutations (Supplementary Fig. S1b, c). We defined the difference between the highest frequency value of each mutation in the early four VOCs (Alpha, Beta, Gamma, Delta) and the highest frequency of Omicron BA.1-5 as the frequency change (*Δ*Frequency). Among them, 13 mutations exhibited increased frequency, while 71 mutations showed decreased frequency (Fig. 1a; Supplementary Fig. S1a). The cell lethality was further measured. The cell lethality was calculated through the ratio of the normalized cell death rate to the protein expression level (Fig. 1b, c; Supplementary Fig. S2). In comparison with the wild-type (WT) 2-E, 13 mutations introduced a stronger capability of killing cells, while 51 mutations attenuated the capability (Fig. 1c).

**Fig. 1:**
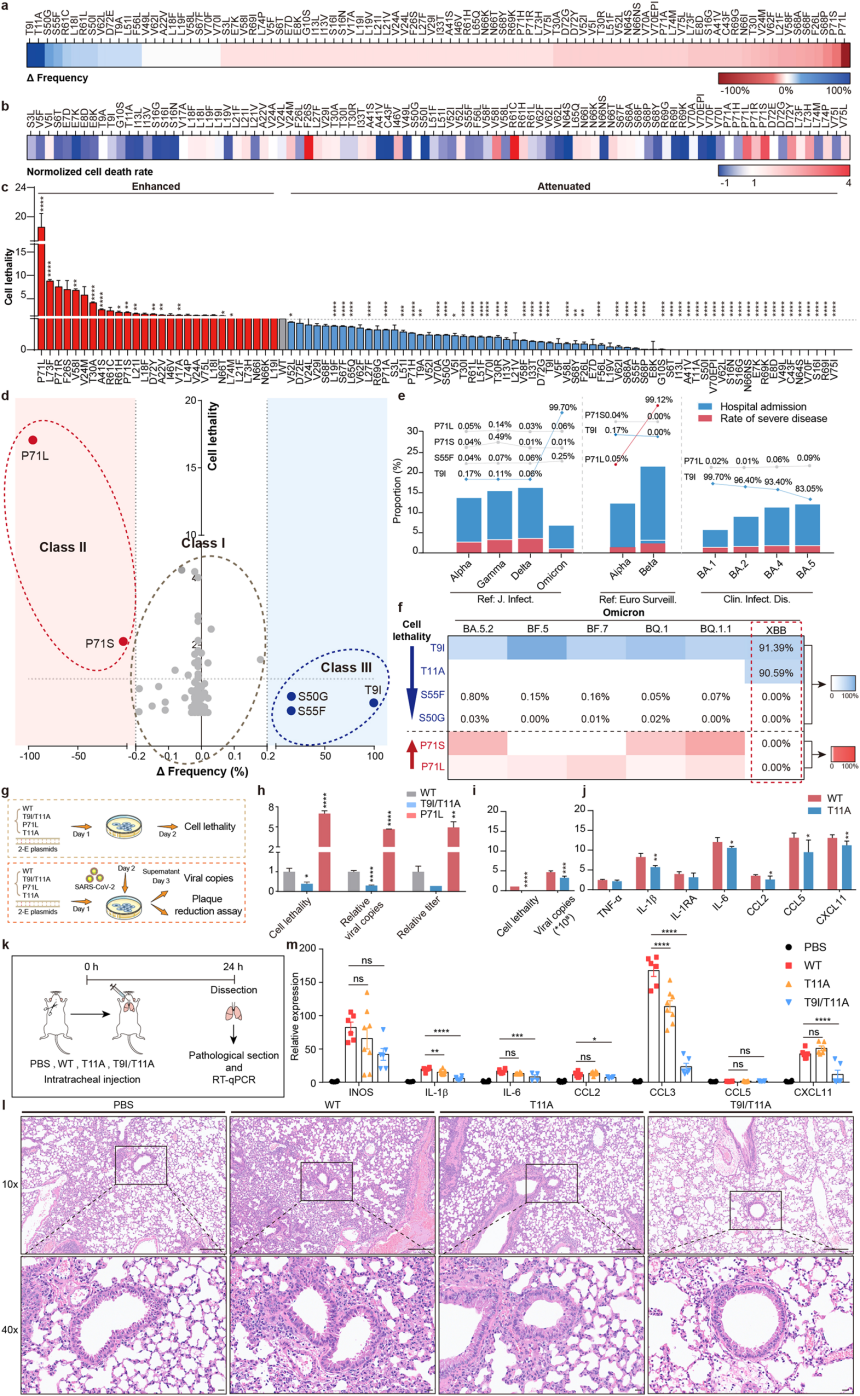
SARS-CoV-2 2-E mutations are potential pathogenicity markers. **a** The *Δ*frequency of 92 2-E mutations. **b** The normalized cell death rate of 2-E mutations. **c** The cell lethality of 2-E mutations. **d** Correlation analysis of *Δ*frequency and cell lethality. The dotted gray circles represent Class I, the dotted red circles represent Class II, and the dotted blue circles represent Class III (Spearman’s correlation analysis: R^2^ = 0.33, *P* < 0.0001). **e** The quantification of hospitalization rate and disease severity up to Omicron BA.5 and the contribution of Class II and III mutations in tabulate. Pathogenicity of different SARS-CoV-2 variants^3,7,8^. **f** Heatmap of 6 key mutation frequencies in Omicron subvariants. **g** Flow chart of the experiments. **h** Cell lethality and viral loads for Vero E6 cells after transfection with plasmids as indicated. **i**, **j** The activity of 2-E WT and T11A in causing cell lethality, cytokine release, and viral production. **k** Flow chart of the experiments. **l** Histopathology of lungs from the 2-E WT, T11A and T9I/T11A protein treatment groups. Scale bars, 10 μm. **m** qRT‒PCR analysis of cytokine levels 24 h after treatment. *P < 0.05; **P < 0.01; ***P < 0.001*;* unpaired Student’s *t*-test. All error bars are SEM (*n* ≥ 3).

Next, we analyzed the correlation between cell lethality and the *Δ*Frequency of each mutation. All mutations could be distinguished into three groups, which were named Class I, Class II and Class III (Fig. 1d). Class I contained 87 mutations with *Δ*Frequency ranging from –0.20% to 0.20%. Class II contained two mutations, P71L and P71S, which significantly increased cell lethality. The remaining 3 mutations, S50G, S55F, and T9I, exhibiting increased frequency and reduced cell lethality, were classified into Class III. Cell lethality and cytokine storm are key determinants of COVID-19 severity^6^. We selected two mutations, P71L from Class II and T9I from Class III, and further evaluated the inflammatory secretion levels. In comparison with WT 2-E, T9I caused significantly lower release level of cytokines and chemokines, while P71L induced higher inflammatory cytokine secretion (Supplementary Fig. S3). In general, the *Δ*Frequency appeared to be negatively correlated with cell lethality and inflammatory levels (Fig. 1d), which inspired us to further explore the correlation between 2-E mutations and virus pathogenicity.

According to three independent clinical studies, we quantified the hospitalization rate and disease severity of Omicron BA.5 infection and tabulated the contribution of the above listed mutations. The results suggested that the mutations in Class II and III are perhaps the essential factors for disease severity. First, Class III (less lethal) mutations appeared more often in the milder variants than in severe variants (Fig. 1e, left). The most representative mutation was T9I, which sharply increased to 99.70% in milder BA.1 and was highly conserved in Omicron (Supplementary Table S1)^7^. Second, Class II (more lethal) mutations were correlated with more severe variants (Fig. 1e, middle). Mutation P71L appeared in Alpha and the frequency increased to 99.12% in Beta, the most severe variant thus far^8^. Third, slight frequency changes in T9I (Class III) and P71L (Class II) mutations may affect virus pathogenicity (Fig. 1e, right). Compared with Omicron BA.1 and BA.2, the clinical symptoms of BA.4 and BA.5 are more severe^3^. Correspondingly, the frequency of T9I dropped 4.30% and 16.65% in the latter two subvariants, respectively. The more lethal P71L increased gradually. These results highlighted the important roles of 2-E mutations in determining virus pathogenicity. We proposed that the five mutations may act as pathogenicity markers of SARS-CoV-2.

Following BA.5, various new subvariants appeared in Omicron. We then supervised the five potential pathogenicity markers in the latest six subvariants before December 2022 (Supplementary Table S2). Among them, BA.5.2, BF.5, BF.7 and BQ.1 were derived from the Omicron BA.5 branch^9^. We found that the T9I mutation was still conserved in these subvariants. On December 20, 2022, the Chinese Center for Disease Control and Prevention announced that XBB was a new variant branch of Omicron that had been imported into China. Although XBB is known as the “strongest immune escape variant”, its pathogenicity remains unclear^10^. Encouragingly, we found that XBB retained the mutation T9I and, notably, gained a new mutation T11A (Fig. 1f). Whether this additional mutation will introduce significant changes to T9I was evaluated first. We found that although the expression level of mutant T9I/T11A far exceeded the expression level of WT (Supplementary Fig. S3a), the double mutation T9I/T11A caused lower cell lethality than WT (Fig. 1g, h). In addition, pre-expression of mutant T9I/T11A significantly attenuated SARS-CoV-2 production compared with WT, as T9I alone did (Fig. 1g, h). Notably, the highly toxic mutation P71L caused stronger cell lethality, higher viral loads and titers than WT (Fig. 1g, h). We further deciphered the roles of T11A in virus pathogenicity. Our previous studies demonstrated that T11A is a dominant-negative mutation of channel function^4^. In comparison with the WT 2-E protein, T11A expression significantly alleviated cell death and caused less cytokine release. The capability of producing virus was also weakened (Fig. 1i, j; Supplementary Fig. S3). The influence of T11A was further evaluated in vivo. C57BL/6 mice were injected with different mutant proteins via intratracheal injection (Fig. 1k). We observed marked inflammatory cell infiltration, edema, pulmonary interstitial hyperemia, hemorrhage and alveolar collapse in the 2-E protein treatment group. In contrast, severe damages in the mutant T11A, T9I/T11A and buffer solution groups were not observed (Fig. 1l). In comparison with the 2-E treatment group, the expression levels of cytokines and chemokines were much lower in T11A, T9I/T11A and TBS treatment groups (Fig. 1m). The same results were obtained in 2-E mutation injury model via tail vein injection (Supplementary Fig. S4). Notably, intratracheal injection of 2-E caused local inflammation only (Supplementary Fig. S5). These clues implied a further weakened pathogenicity of the XBB subvariant.

Predicting and rapidly characterizing the virus pathogenicity of new variants is critical for assessing disease dynamics. Multiple viral proteins could be involved in pathogenicity, such as S, non-structural protein 6 (NSP6), etc^11^. In this study, five 2-E mutations were proposed to be potential pathogenicity markers. We applied our predictive theoretical model to forecast the potential pathogenicity of XBB. Two high-frequency mutations with reduced cell lethality were observed, which might confer weaker pathogenicity to XBB. Nevertheless, we still need to be vigilant regarding whether there will be a sudden increase in the frequency of highly pathogenic mutations. There is often an evolutionary trade-off mode between virulence and transmissibility, which could help the virus to achieve optimal fitness^12^. Although the literatures on SARS-CoV-2 transmission, immune escape and evolutionary analysis are vast, we believe that this study provides critical information for epidemic prevention of COVID-19. Preliminary exploration revealed that ubiquitination and degradation might affect the expression level of 2-E mutations (Supplementary Fig. S6). Interestingly, it has also been found that the 2-E protein-related ubiquitination enzyme RING finger protein 5 (RNF5) is closely related to the severity of the disease caused by SARS-CoV-2^13^. In addition, as the innate immune response is closely associated with pathogenicity, the effects of E mutants on the innate antiviral response need further investigation in the future^14^. The analyses performed here come with limitations. First, the influence of the proposed mutations needs to be verified at the virus level by reverse genetics systems. Second, the data linking the genomic sequencing results of 2-E and clinical patient severity are lacking. Nevertheless, as some countries are at the peak of infections, our findings might provide advance warning of potential outbreaks.

## Supplementary information

Supplementary Information

## References

[1] Cao Y (2023). Nature.

[2] Suzuki R (2022). Nature.

[3] Morris CP (2023). Clin. Infect. Dis..

[4] Xia B (2021). Cell Res..

[5] Xia B (2022). Innovation.

[6] Lee S (2020). Trends Immunol..

[7] Esper FP (2023). J. Infect. Dis..

[8] Funk T (2021). Eur. Surveill..

[9] Sun Y (2023). J. Biosaf. Biosecur..

[10] Wang Q (2023). Cell.

[11] Chen DY (2023). Nature.

[12] Geoghegan JL (2018). Nat. Rev. Genet..

[13] Li Z (2023). Signal Transduct. Target. Ther..

[14] Zheng M (2021). Nat. Immunol..

