# Supplementary material for: Impact of SARS-CoV-2 envelope protein mutations on the pathogenicity of Omicron XBB
Source: Cell Discov. 2023 Jul 28;9:80. doi: 10.1038/s41421-023-00575-7 (PMC10382526; doi:10.1038/s41421-023-00575-7)
Supplement: Supplementary file 1 — Supplementary Information [file 41421_2023_575_MOESM1_ESM.pdf]

Supplementary figure and tables:

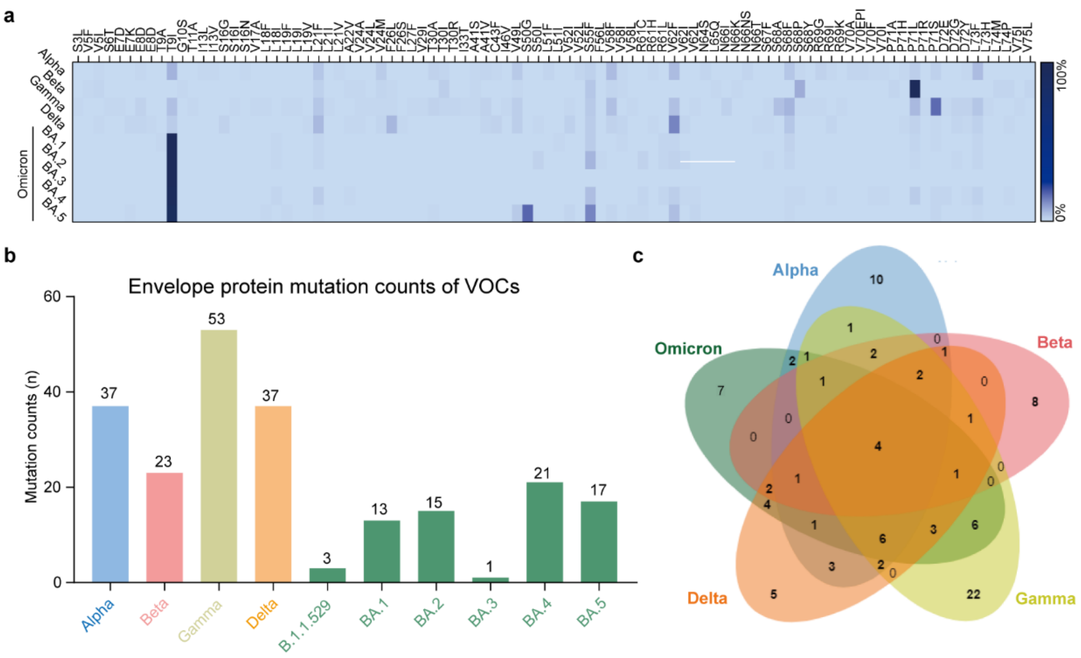

Fig.S1 Statistics of 2-E mutations. a. Heat map of 2-E mutations frequency in five VOCs. **b.** The mutation counts of 2-E in each VOCs. **c.** Venn diagram of 2-E mutations in Omicron compared with other VOCs.

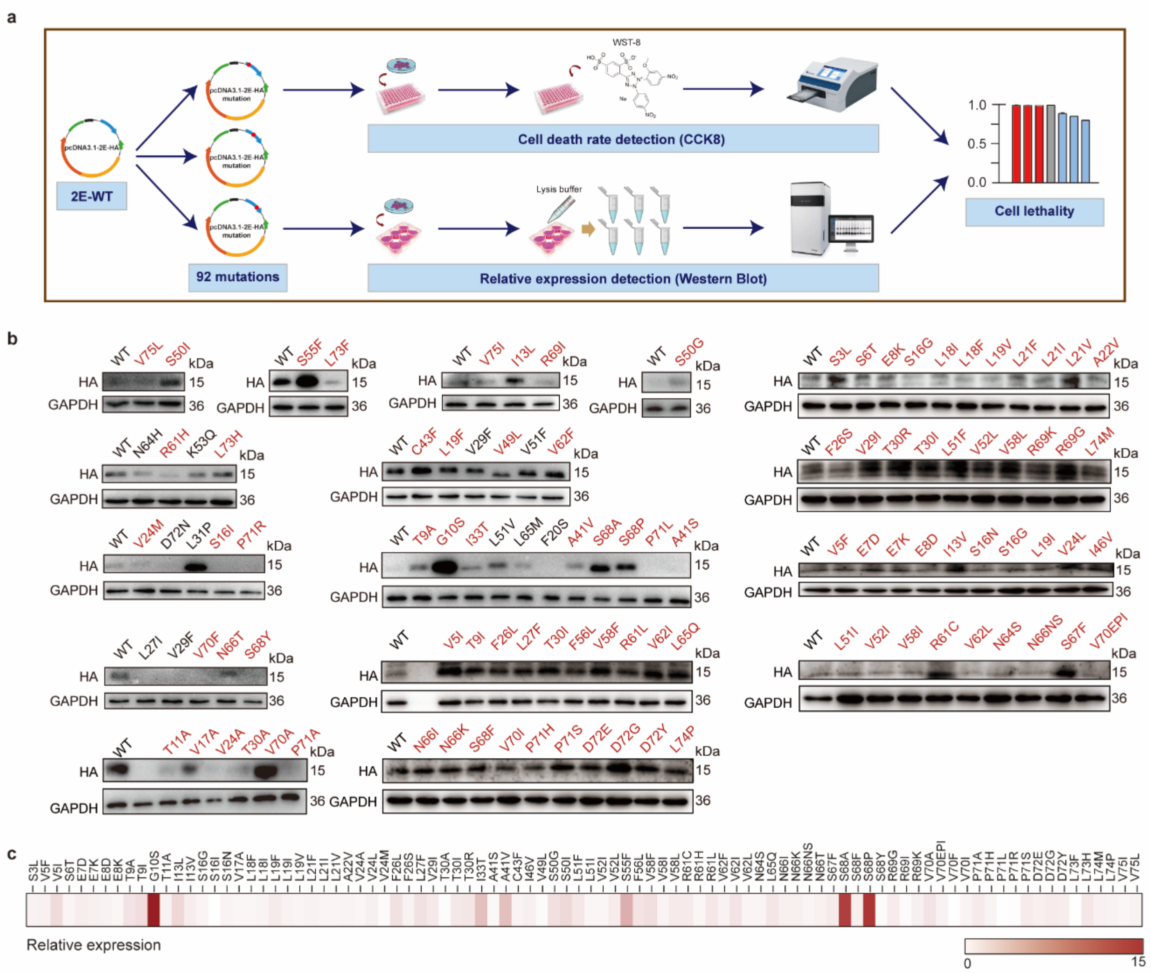

Fig.S2 Cell lethality detection of 2-E mutations. a. Schematic of cell lethality detection. b and c. The protein expression level of 2-E mutations in Vero E6 cells after transfection.

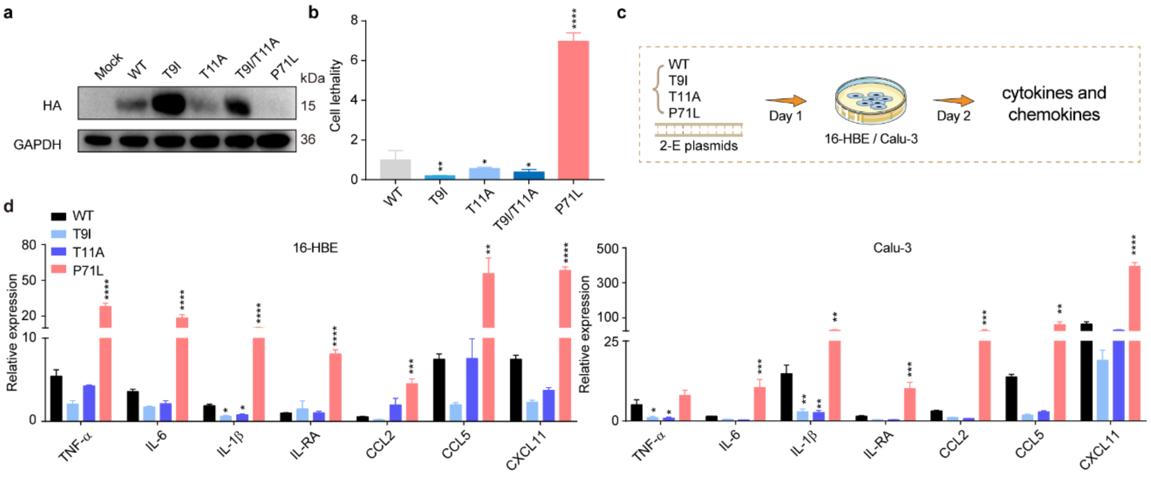

Fig.S3 T9I and T11A weaken cell lethality and inflammation.

a， Protein expression levels of WT ,T9I/T11A or P71L in Vero E6 cells after transfection. **b,** The cell lethality for Vero E6 cells after transfection with plasmids as indicated. **c,** Transfection of Calu-3 and 16HBE cells. **d,** Expression of cytokines and chemokines following transfection of WT, T9I T11A and P71L plasmids, measuring mRNA expression via qRT-PCR. All data are representative of three independent experiments. *$^*p < 0.05$; $^{**}p < 0.01$; $^{***}p < 0.001$;* unpaired Student’s t test. All error bars are SEM (n⩾3).

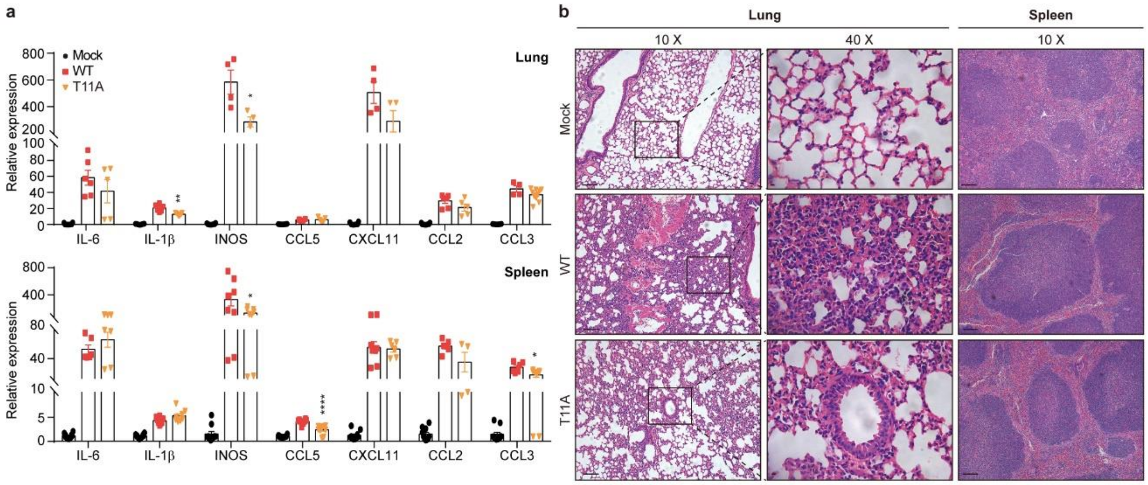

Fig.S4 T11A cause less severe inflammation than wild type 2-E channel *in vivo*.

a, qRT-PCR analysis of cytokine levels after 24 h treatment. **b,** Histopathology of lungs and spleens from 2-E WT and T11A proteins treatment groups (bar, 100 μm). *$^*p < 0.05$; $^{**}p < 0.01$; $^{***}p < 0.001$;* unpaired Student’s t test. All error bars are SEM (n⩾3).

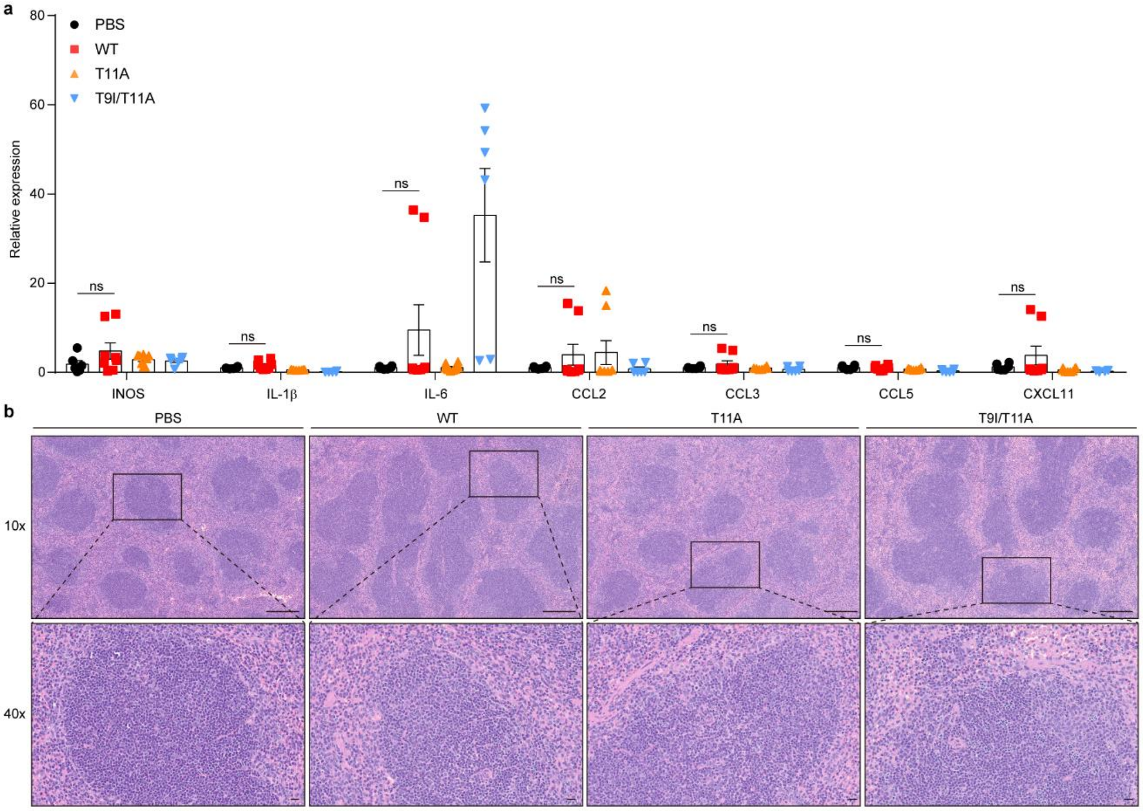

Fig.S5 Intratracheal injection of 2-E did not cause severe inflammation in spleen.

a, qRT-PCR analysis of cytokines and chemokines levels after 24 h treatment. *$p < 0.05$; **$p < 0.001$; unpaired Student's t test. All error bars are SEM. **b,** Histopathology of spleens from PBS, WT, T11A and T9I/T11A proteins treatment groups (bar, 10 μm).

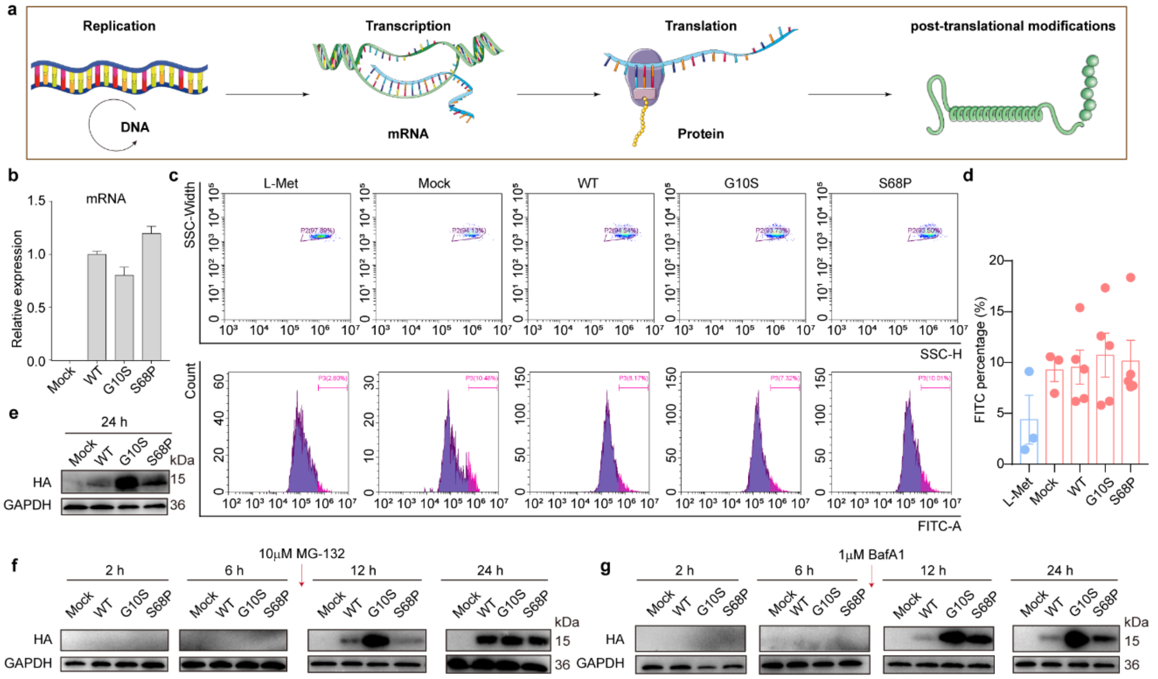

Fig.S6 Ubiquitination and degradation of 2-E protein affect its expression level.

a, Flow chart of the experiments. Potential mechanisms affecting 2-E protein expression level were explored at different levels. **b,** Vero E6 cells transfected with WT, G10S, and S68P. The RNA of cells was extracted after 24 hours and examined by qRT-PCR. The mRNA level of 2-E mutations as indicated. **c and d,** The overall translation efficiency detection of 2-E mutations as indicated. **e,** The protein expression level of 2-E mutations as indicated. **f-g,** The expression level of 2-E mutations at different time points under 10 μM MG132 (f) and 1 μM BafA1 treatment. **p < 0.05; **p < 0.001*; unpaired Student's t test. All error bars are SEM.

Supplementary Table S1 2-E mutations with frequency ≥ 0.01% in five VOC

					Omicron				
	**Alpha**	**Beta**	**Gamma**	**Delta**	**BA.1**	**BA.2**	**BA.3**	**BA.4**	**BA.5**
**S3L**	0.00%	0.00%	0.01%	0.00%	0.00%	0.00%	0.00%	0.00%	0.00%
**V5F**	0.00%	0.01%	0.00%	0.00%	0.00%	0.00%	0.00%	0.00%	0.00%
**V5I**	0.01%	0.02%	0.00%	0.01%	0.00%	0.00%	0.00%	0.00%	0.00%
**S6T**	0.00%	0.00%	0.01%	0.00%	0.00%	0.00%	0.00%	0.00%	0.00%
**E7D**	0.00%	0.00%	0.01%	0.00%	0.00%	0.00%	0.00%	0.00%	0.00%
**E7K**	0.00%	0.00%	0.00%	0.02%	0.00%	0.00%	0.00%	0.00%	0.00%
**E8D**	0.01%	0.00%	0.03%	0.02%	0.00%	0.00%	0.00%	0.00%	0.00%
**E8K**	0.00%	0.00%	0.00%	0.01%	0.00%	0.00%	0.00%	0.00%	0.00%
**T9A**	0.00%	0.00%	0.00%	0.00%	0.01%	0.00%	0.00%	0.00%	0.00%
**T9I**	0.17%	0.00%	0.11%	0.06%	99.70%	96.39%	99.50%	93.47%	83.07%
**G10S**	0.00%	0.01%	0.00%	0.00%	0.00%	0.00%	0.00%	0.00%	0.00%
**T11A**	0.00%	0.00%	0.01%	0.00%	0.00%	0.00%	0.00%	0.00%	0.00%
**I13L**	0.01%	0.00%	0.00%	0.00%	0.00%	0.00%	0.00%	0.00%	0.00%
**I13V**	0.01%	0.00%	0.00%	0.00%	0.00%	0.00%	0.00%	0.00%	0.00%
**S16G**	0.00%	0.00%	0.00%	0.03%	0.00%	0.00%	0.00%	0.00%	0.00%
**S16I**	0.00%	0.00%	0.01%	0.00%	0.00%	0.00%	0.00%	0.00%	0.00%
**S16N**	0.01%	0.00%	0.00%	0.00%	0.00%	0.00%	0.00%	0.00%	0.00%
**V17A**	0.00%	0.00%	0.01%	0.00%	0.00%	0.00%	0.00%	0.00%	0.00%
**L18F**	0.00%	0.00%	0.01%	0.00%	0.00%	0.00%	0.00%	0.00%	0.00%
**L18I**	0.00%	0.00%	0.00%	0.00%	0.01%	0.01%	0.00%	0.00%	0.00%
**L19F**	0.00%	0.00%	0.01%	0.00%	0.00%	0.00%	0.00%	0.00%	0.00%
**L19I**	0.00%	0.00%	0.01%	0.00%	0.00%	0.00%	0.00%	0.00%	0.00%
**L19V**	0.00%	0.00%	0.00%	0.01%	0.00%	0.00%	0.00%	0.00%	0.00%
**L21F**	0.08%	0.04%	0.08%	0.14%	0.02%	0.02%	0.00%	0.00%	0.00%

					Omicron				
	**Alpha**	**Beta**	**Gamma**	**Delta**	**BA.1**	**BA.2**	**BA.3**	**BA.4**	**BA.5**
**L21I**	0.00%	0.00%	0.01%	0.00%	0.00%	0.00%	0.00%	0.00%	0.00%
**L21V**	0.01%	0.00%	0.00%	0.00%	0.00%	0.00%	0.00%	0.00%	0.00%
**A22V**	0.00%	0.01%	0.00%	0.01%	0.00%	0.00%	0.00%	0.00%	0.00%
**V24A**	0.00%	0.01%	0.01%	0.01%	0.00%	0.00%	0.00%	0.00%	0.00%
**V24L**	0.00%	0.00%	0.01%	0.00%	0.00%	0.00%	0.00%	0.00%	0.00%
**V24M**	0.06%	0.02%	0.01%	0.02%	0.00%	0.00%	0.00%	0.00%	0.00%
**F26L**	0.00%	0.01%	0.01%	0.15%	0.00%	0.00%	0.00%	0.00%	0.00%
**F26S**	0.00%	0.00%	0.00%	0.01%	0.00%	0.00%	0.00%	0.00%	0.00%
**L27F**	0.00%	0.00%	0.01%	0.00%	0.00%	0.00%	0.00%	0.00%	0.00%
**V29I**	0.00%	0.00%	0.01%	0.00%	0.00%	0.00%	0.00%	0.00%	0.00%
**T30A**	0.03%	0.00%	0.03%	0.00%	0.00%	0.00%	0.00%	0.00%	0.00%
**T30I**	0.02%	0.05%	0.02%	0.00%	0.00%	0.00%	0.00%	0.00%	0.00%
**T30R**	0.00%	0.00%	0.02%	0.00%	0.00%	0.00%	0.00%	0.00%	0.00%
**I33T**	0.00%	0.00%	0.01%	0.00%	0.00%	0.00%	0.00%	0.00%	0.00%
**A41S**	0.01%	0.01%	0.01%	0.00%	0.00%	0.00%	0.00%	0.00%	0.00%
**A41V**	0.02%	0.00%	0.03%	0.02%	0.00%	0.00%	0.00%	0.00%	0.00%
**C43F**	0.01%	0.00%	0.00%	0.03%	0.00%	0.00%	0.00%	0.00%	0.00%
**I46V**	0.00%	0.00%	0.01%	0.00%	0.00%	0.00%	0.00%	0.00%	0.00%
**V49L**	0.07%	0.01%	0.05%	0.05%	0.00%	0.00%	0.00%	0.00%	0.00%
**S50G**	0.00%	0.00%	0.00%	0.00%	0.00%	0.00%	0.00%	0.00%	0.51%
**S50I**	0.00%	0.00%	0.00%	0.01%	0.00%	0.03%	0.00%	0.00%	0.00%
**L51F**	0.02%	0.00%	0.00%	0.00%	0.00%	0.00%	0.00%	0.00%	0.00%
**L51I**	0.00%	0.00%	0.00%	0.00%	0.01%	0.00%	0.00%	0.00%	0.00%
**V52I**	0.00%	0.00%	0.01%	0.04%	0.00%	0.00%	0.00%	0.00%	0.00%
**V52L**	0.02%	0.00%	0.00%	0.00%	0.00%	0.00%	0.00%	0.00%	0.00%
**S55F**	0.04%	0.08%	0.07%	0.06%	0.25%	0.25%	0.10%	0.15%	0.21%

					Omicron				
	Alpha	Beta	Gamma	Delta	BA.1	BA.2	BA.3	BA.4	BA.5
**F56L**	0.00%	0.00%	0.00%	0.00%	0.00%	0.01%	0.00%	0.00%	0.00%
**V58F**	0.13%	0.02%	0.11%	0.03%	0.00%	0.00%	0.00%	0.00%	0.00%
**V58I**	0.01%	0.01%	0.00%	0.02%	0.01%	0.00%	0.00%	0.00%	0.00%
**V58L**	0.00%	0.00%	0.01%	0.00%	0.00%	0.00%	0.00%	0.00%	0.00%
**V61C**	0.00%	0.00%	0.00%	0.00%	0.00%	0.03%	0.00%	0.00%	0.00%
**V61H**	0.01%	0.00%	0.00%	0.01%	0.00%	0.00%	0.00%	0.00%	0.00%
**V61L**	0.00%	0.00%	0.00%	0.00%	0.02%	0.02%	0.00%	0.00%	0.00%
**V62F**	0.13%	0.00%	0.11%	0.40%	0.02%	0.03%	0.00%	0.00%	0.32%
**V62I**	0.00%	0.00%	0.00%	0.02%	0.00%	0.02%	0.00%	0.00%	0.00%
**V62L**	0.00%	0.00%	0.01%	0.00%	0.00%	0.00%	0.00%	0.00%	0.00%
**N64S**	0.02%	0.00%	0.00%	0.00%	0.00%	0.00%	0.00%	0.00%	0.00%
**L65Q**	0.00%	0.01%	0.00%	0.00%	0.00%	0.00%	0.00%	0.00%	0.00%
**N66I**	0.00%	0.00%	0.04%	0.00%	0.00%	0.00%	0.00%	0.00%	0.00%
**N66K**	0.00%	0.01%	0.00%	0.00%	0.00%	0.00%	0.00%	0.00%	0.00%
**N66NS**	0.02%	0.00%	0.00%	0.00%	0.00%	0.00%	0.00%	0.00%	0.00%
**N66T**	0.00%	0.00%	0.00%	0.01%	0.00%	0.00%	0.00%	0.00%	0.00%
**S67F**	0.00%	0.00%	0.01%	0.01%	0.00%	0.01%	0.00%	0.00%	0.00%
**S68A**	0.00%	0.00%	0.11%	0.00%	0.00%	0.00%	0.00%	0.00%	0.00%
**S68F**	0.08%	0.00%	0.16%	0.08%	0.03%	0.02%	0.00%	0.00%	0.00%
**S68P**	0.01%	0.20%	0.01%	0.00%	0.00%	0.00%	0.00%	0.00%	0.00%
**S68Y**	0.00%	0.00%	0.01%	0.00%	0.00%	0.00%	0.00%	0.00%	0.00%
**R69G**	0.03%	0.00%	0.01%	0.00%	0.00%	0.00%	0.00%	0.00%	0.00%
**R69I**	0.01%	0.00%	0.00%	0.02%	0.00%	0.00%	0.00%	0.00%	0.00%
**R69K**	0.00%	0.00%	0.01%	0.00%	0.00%	0.00%	0.00%	0.00%	0.00%
**V70A**	0.02%	0.00%	0.00%	0.00%	0.00%	0.00%	0.00%	0.00%	0.00%
**V70EPI**	0.02%	0.00%	0.00%	0.00%	0.00%	0.00%	0.00%	0.00%	0.00%

					Omicron				
	**Alpha**	**Beta**	**Gamma**	**Delta**	**BA.1**	**BA.2**	**BA.3**	**BA.4**	**BA.5**
**V70F**	0.01%	0.00%	0.00%	0.00%	0.00%	0.00%	0.00%	0.00%	0.00%
**V70I**	0.01%	0.00%	0.00%	0.00%	0.00%	0.00%	0.00%	0.00%	0.00%
**P71A**	0.00%	0.02%	0.00%	0.00%	0.00%	0.00%	0.00%	0.00%	0.00%
**P71H**	0.01%	0.00%	0.00%	0.01%	0.00%	0.00%	0.00%	0.00%	0.00%
**P71L**	0.05%	99.12%	0.14%	0.03%	0.02%	0.01%	0.00%	0.06%	0.09%
**P71R**	0.00%	0.00%	0.01%	0.00%	0.00%	0.00%	0.00%	0.00%	0.00%
**P71S**	0.04%	0.00%	0.49%	0.01%	0.01%	0.01%	0.00%	0.01%	0.01%
**D72E**	0.00%	0.00%	0.01%	0.00%	0.00%	0.00%	0.00%	0.00%	0.00%
**D72G**	0.00%	0.00%	0.03%	0.02%	0.00%	0.01%	0.00%	0.00%	0.00%
**D72Y**	0.01%	0.00%	0.03%	0.01%	0.00%	0.00%	0.00%	0.00%	0.00%
**L73F**	0.13%	0.00%	0.09%	0.04%	0.02%	0.02%	0.00%	0.05%	0.01%
**L73H**	0.00%	0.00%	0.01%	0.00%	0.00%	0.00%	0.00%	0.00%	0.00%
**L74M**	0.00%	0.02%	0.00%	0.00%	0.00%	0.00%	0.00%	0.00%	0.00%
**L74P**	0.00%	0.02%	0.00%	0.01%	0.00%	0.00%	0.00%	0.00%	0.00%
**V75I**	0.00%	0.00%	0.01%	0.00%	0.00%	0.00%	0.00%	0.00%	0.00%
**V75L**	0.00%	0.02%	0.00%	0.00%	0.00%	0.00%	0.00%	0.00%	0.00%

Supplementary Table S2 2-E mutations with frequency ≥ 0.01% in newly emergent Omicron sub-variants

	**BA.5.2**	**BF.5**	**BF.7**	**BQ.1**	**BQ.1.1**	**XBB**
**V5I**	0.01%	0.01%	0.11%	0.01%	0.01%	0.00%
**E7K**	0.01%	0.01%	0.00%	0.00%	0.00%	0.00%
**E8D**	0.00%	0.00%	0.00%	0.00%	0.01%	0.00%
**T9A**	0.01%	0.01%	0.00%	0.01%	0.00%	0.00%
**T9I**	91.68%	96.76%	91.44%	93.13%	91.19%	91.39%

**T11A**	0.00%	0.00%	0.00%	0.00%	0.01%	90.59%
**S16G**	0.00%	0.00%	0.00%	0.00%	0.01%	0.00%
**L18I**	0.04%	0.01%	0.05%	0.00%	0.00%	0.00%
**L21F**	0.03%	0.01%	0.03%	0.06%	0.02%	0.00%
**L21I**	0.00%	0.00%	0.03%	0.00%	0.00%	0.00%
**A22V**	0.00%	0.00%	0.00%	0.01%	0.00%	0.00%
**V24L**	0.01%	0.00%	0.00%	0.00%	0.00%	0.00%
**V24M**	0.01%	0.00%	0.00%	0.02%	0.01%	0.00%
**F26L**	0.01%	0.00%	0.00%	0.01%	0.00%	0.00%
**T30I**	0.00%	0.00%	0.00%	0.00%	0.01%	0.00%
**A41V**	0.01%	0.00%	0.00%	0.01%	0.00%	0.00%
**C43F**	0.00%	0.00%	0.00%	0.01%	0.00%	0.00%
**V49L**	0.01%	0.01%	0.00%	0.00%	0.00%	0.00%
**S50G**	0.03%	0.00%	0.01%	0.02%	0.00%	0.00%
**S50I**	0.01%	0.05%	0.00%	0.00%	0.00%	0.00%
**L51F**	0.00%	0.00%	0.00%	0.00%	0.00%	0.00%
**L51I**	0.01%	0.01%	0.00%	0.00%	0.02%	0.00%
**S55F**	0.80%	0.15%	0.16%	0.79%	0.05%	0.00%
**V58F**	0.01%	0.00%	0.00%	0.00%	0.00%	0.00%
**V58I**	0.01%	0.03%	0.00%	0.01%	0.01%	0.00%
**V58L**	0.02%	0.00%	0.00%	0.00%	0.02%	0.00%
**R61C**	0.01%	0.00%	0.00%	0.00%	0.00%	0.00%
**R61L**	0.01%	0.00%	0.19%	0.00%	0.00%	0.00%
**V62F**	0.27%	0.07%	0.21%	0.01%	0.06%	0.00%
**V62I**	0.01%	0.04%	0.00%	0.00%	0.00%	0.00%
**N66K**	0.00%	0.01%	0.01%	0.00%	0.00%	0.00%
**S68F**	0.04%	0.06%	0.00%	0.02%	0.00%	0.00%
**S68P**	0.00%	0.02%	0.02%	0.01%	0.00%	0.00%

**V70A**	0.00%	0.00%	0.01%	0.00%	0.00%	0.00%
**V70I**	0.00%	0.00%	0.00%	0.00%	0.01%	0.00%
**P71H**	0.01%	0.00%	0.01%	0.00%	0.00%	0.00%
**P71L**	0.02%	0.03%	0.05%	0.02%	0.04%	0.00%
**P71S**	0.09%	0.00%	0.00%	0.07%	0.11%	0.00%
**D72G**	0.01%	0.13%	0.01%	0.00%	0.01%	0.00%
**D72H**	0.00%	0.00%	0.00%	0.00%	0.00%	0.18%
**L73F**	0.01%	0.02%	0.05%	0.01%	0.01%	0.09%

Methods

Mutation frequency statistics

The frequency of 2-E spontaneous mutations was collected from National Genomics Data Center (NGDC). All mutations with frequency ≥ 0.01% were selected to further analysis. The ⊿frequency was defined as difference of the maximum frequency value of early VOCs 2-E mutations and that of Omicron sub-variants.

Plasmids and mutagenesis

Wild-type SARS-CoV-2-E sequences were synthesized in pcDNA3.1 with a HA tag by the Beijing Genomics Institute (BGI, China). Point mutations were generated using sited-directed mutagenesis, and all mutations were confirmed by sequencing (BGI, China).

Cell culture

Vero E6 cells were purchased from National Collection of Authenticate Cell Cultures (China). Vero E6 cells were grown in 90% DMEM basal medium (Gibco, NY, USA) supplemented with 10% fetal bovine serum (Gibco, NY, USA) and 100 units/mL penicillin/streptomycin (Gibco, NY, USA). Cells were grown at 37 °C, 5% CO_2 incubator, and passaged approximately every 2 days when on fluency up to 80%–90%. Vero-E6 cells were seeded on the 6-well cell culture plates for western blot and 96-well culture plates for CCK-8 assay.

Western blot

Cells were lysed by lysis buffer (20118ES60, Yeasen, China). Then the proteins were collected and quantified by BCA protein assay kit (Thermofisher, USA). Proteins were resolved in 12% SDS-PAGE, transferred to PVDF membranes (GE, USA), and incubated with primary antibodies against HA-Tag (proteintech, USA), GAPDH (Yeasen, China). Second antibodies are peroxidase-Conjugated Goat Anti-Rabbit IgG (H+L) (33101ES60, Yeasen, China) and Peroxidase AffiniPure Goat Anti-Mouse IgG (H+L) (33201ES60, Yeasen, China). Then the relative expressions of 2-E mutations were analyzed with ImageJ.

Cell death rate assay

Vero E6 cells were transfected with 400 ng/ well 2-E plasmids or 2-E mutations using Lipofectamine 3000 Transfection Reagent (L3000015, Thermo Fisher, MA, USA). The control group was Vero E6 cells transfected with 400 ng/ well pcDNA3.1 vector plasmids alone. After 24 h, we tested cell viability by CCK-8 kit (40203ES60, Yeasen, China) according to our previous work. Absorbance analysis were performed with Thermo Scientific Microplate Reader at 450 nm according to the manufacturer's instructions (Thermo Fisher, MA, USA). Normalized cell death rate = $(A_{blank} - A_{mutaion})/(A_{blank} - A_{model})$.

Animal model

To evaluate the pathogenicity of 2-E mutations *in vivo*, 2-E mutation proteins was conducted through intratracheal injection (2.5 mg/kg body weight) and tail vein injection (25 mg/kg body weight) in 8-week-old mice to establish mice model. Male C57BL/6 mice were obtained from Shanghai SLAC Laboratory Animal Co., Ltd. All animal procedures were performed in accordance with the National Institutes of Health Guide for the Care

and Use of Laboratory Animals, under protocols approved and strictly followed by the Institutional Animal Care and Use Committees (IACUC).

Histology

Tissues from 2-E WT or T11A treated mice were fixed in 4% PFA for at least 7 days, and then were paraffin embedded. The paraffin samples were cut into 3-μm sections following the standard procedure. The sections were stained with H&E, and examined by Leica TCS-SP8 STED system (Leica Microsystems, DE).

Viral release assay

SARS-CoV-2 (nCoV-2019BetaCoV/Wuhan/WIV04/2019) was preserved at Wuhan Institute of Virology, Chinese Academy of Sciences (CAS). Its associated operations were performed in a biosafety level 3 (BSL-3) facility. For viral release assay, Vero E6 cells seeded in 48-well plate with 50,000 cells per well overnight were transfected with 2-E WT or mutations at day 1, than infected with SARS-CoV-2 (MOI=0.01) at day 2. At day 3, viral RNA was extracted from cell supernatants with Mini BEST Viral RNA/DNA Extraction Kit (Takara, Japan) according to the instructions, then reverse transcribed with Prime ScriptTM RT reagent Kit with gDNA Eraser (Takara, Japan). Viral genome copies were quantified with Takara TB Green® Premix Ex Taq™ II (Takara, Japan) by a standard curve method on ABI 7500 using a pair of primers targeting S gene. The forward primer: 5′-CAATGGTTTAACAGGCACAGG -3′, the reverse primer: 5′-CTCAAGTGTCTGTGGATCACG-3′.

qRT-PCR Analysis

Total RNAs were extracted from cells or tissues using Trizol (Invitrogen, USA) and all total Nucleic Acid Isolation Kit (Ambion Inc., USA), following the manufacturer's instruction. The experiment was performed using SYBR Green Master Mix (11184ES03, Yeasen, China) to quantify the mean values of delta Ct and SEM (n≥ 3). The primers used for quantification were listed in Supplementary information, Supplementary Table S3.

Supplementary Table S3: List of Quantitative Real-time PCR (qRT-PCR) primers.

Primer Name	FWD sequence	REV sequence
CCL2	AGGTCCCTGTCATGCTTCTG	TCTGGACCCATTCCTTCTTG
CCL3	ATGAAGGTCTCCACCACTGC	CCCAGGTCTCTTTGGAGTCA
CCL5	CCCTCACCATCATCCTCACT	CCTTCGAGTGACAAACACGA
IL-6	AGTTGCCTTCTTGGGACTGA	TCCACGATTTCCCAGAGAAC
IL-1RA	CCAGCTCATTGCTGGGTACT	TTCTCAGAGCGGATGAAGGT
IL-1β	GAAGTTGACGGACCCCAAAA	CCACAGCCACAATGAGTGATAC
CXCL9	GAACGGAGATCAAACCTGCC	CGACGACTTTGGGGTGTTTT
INOS	GTTCTCAGGCCAACAATACAAGA	GTGGACGGGTCGATGTCAC
TNF-α	TCGTAGCAAACCACCAAGTG	GGAGTAGACAAGGTACAACCCA
GAPDH	AACTTTGGCATTGTGGAAGG	ACACATTGGGGGTAGGAACA

Protein synthesis assay

Vero-E6 cells were transfected with 2-E WT, G10S, S68P or mock. After 24 hours, cells were labeled by 1 mM AHA (C10102, Thermofisher, USA) or methionine (HY-N0326, MCE, USA) and treated according to the protocol[1], Alexa Fluor488 alkyne was

bought from Thermofisher (A10267, USA). Dxflex (Beckman Coulter, USA) was used to monitoring vero-E6 cells global protein synthesis after transfection.

Reference

1 Imami, K. & Yasuda, T. Measuring Protein Synthesis during Cell Cycle by Azidohomoalanine (AHA) Labeling and Flow Cytometric Analysis. *Bio Protoc* **9**, e3215, doi:10.21769/BioProtoc.3215 (2019).
